# Estimation of auroral emission altitude in the region of the ISS magnetic footprint during a relativistic electron precipitation event observed by ISS-CALET

**DOI:** 10.1186/s40623-026-02507-7

**Published:** 2026-09-18

**Authors:** Kyutaro Yanagisawa, Ryuho Kataoka, Kanako Seki, Daniel Whiter, Yoshizumi Miyoshi, Kazuo Shiokawa, Martin Connors, Shoji Torii, Satoshi Nakahira, Takeshi Sakanoi, Raju Aryal, Alessandro Bruno, Lauren W. Blum

**Affiliations:** 1 Graduate School of Science, The University of Tokyo, 7-3-1 Hongo, Bunkyo-ku, Tokyo, 113-0033 Japan; 2 Okinawa Institute of Science and Technology, 1919-1 Tancha, Onna, Kunigami District, Okinawa, 904-0495 Japan; 3 Research Center for Advanced Science and Technology (RCAST), The University of Tokyo, 4-6-1 Komaba, Meguro-ku, Tokyo, 153-8904 Japan; 4 Department of Physics and Astronomy, University of Southampton, University Road, Southampton, SO17 1BJ UK; 5 Institute for Space-Earth Environmental Research, Nagoya University, Furo-Cho, Chikusa-ku, Nagoya, Aichi 464-8601 Japan; 6 Department of Physics and Astronomy, Athabasca University, Athabasca, AB T9S 3A3 Canada; 7 Waseda Research Institute for Science and Engineering, Waseda University, 17 Kikui-cho, Shinjuku-ku, Tokyo, 162-0044 Japan; 8 Institute of Space and Astronautical Science (ISAS), Japan Aerospace Exploration Agency (JAXA), 3-1-1 Yoshinodai, Chuo-ku, Sagamihara, Kanagawa 252-5210 Japan; 9 Graduate School of Science, Tohoku University, 6-3 Aramaki-Aza Aoba, Aoba-Ku, Sendai, Miyagi 980-8578 Japan; 10 Heliophysics Science Division, NASA Goddard Space Flight Center, 8800 Greenbelt Rd, Greenbelt, MD 20771 USA; 11 Laboratory for Atmospheric and Space Physics and Department of Aerospace Engineering Sciences, University of Colorado Boulder, 1234 Innovation Drive, Boulder, CO 80303-7814 USA

**Keywords:** Electron precipitation, Atmospheric ionization, Aurora, Stereoscopy, Space weather forecast

## Abstract

We report a multi-MeV relativistic electron precipitation (REP) event as observed by the CALorimetric Electron Telescope and the Monitor of All-sky X-ray Image aboard the International Space Station, together with low-cost twin all-sky auroral imagers installed at Athabasca, Canada. We find that visible auroral emissions near the ISS magnetic footprint during REP events are not frequent—observed in only two of 10 conjugate REP events during our survey period from September 2024 to September 2025—and that one case, on 3 May 2025, featured diffuse aurora near the ISS magnetic footprint. Using two independent stereoscopic methods, we estimate the auroral emission height to be ~90 km, which is typically produced by tens-of-keV electron precipitation. Combined with the MeV electron precipitation detected by CALET, such broadband electron precipitation from tens-of-keV to MeV is consistent with the hypothesis of chorus-driven REP events. The emission altitude shows no systematic latitudinal variation, which is not consistent with field-line curvature scattering type energy-dispersed precipitation. The combined space- and ground-based observations contribute to a better understanding of the spatial context of wave–particle interaction associated with the REP events.

**Graphical Abstract:**

**Figure:**
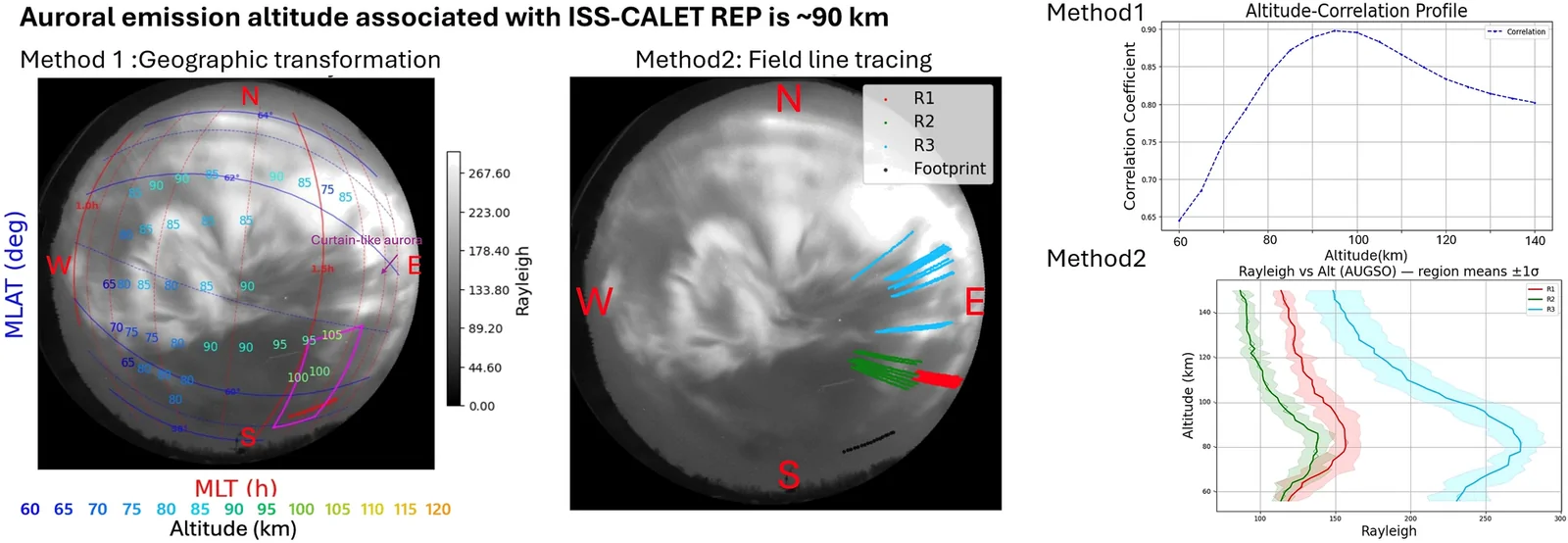

## Introduction

Relativistic electrons with energies of several hundred keV to a few MeV occasionally precipitate into the Earth’s atmosphere with various spatial and temporal scales. Such relativistic electron precipitation (REP) events have been first detected by ground-based observations (Anderson and Milton 1964; Bailey & Pomerantz 1965; Rosenberg et al. 1972) and later by balloon and satellite measurements (Anderson et al. 1968; Imhof et al. 1986; Lorentzen et al. 2000; Nakamura et al. 1995, 2000; Comess et al. 2013; Carson et al. 2013). The importance of REP events can be categorized as: (1) revealing their causes contributes to predictive understanding of the dynamic variation of the outer radiation belt, because the REP events are associated with a major loss process for trapped MeV electrons (Lorentzen et al. 2000; Kubota et al. 2015; Kurita et al. 2018; Millan et al. 2002; Miyoshi et al. 2008, 2015). (2) The REP-related ionization of the middle atmosphere causes the production of NOx and HOx and subsequent ozone destruction (Daae et al. 2012; Isono et al. 2014a,b; Kataoka et al. 2019; Miyoshi et al. 2015, 2021; Turunen et al. 2016; Murase et al. 2022). (3) Radiation dose exposure at LEO, including to the body of astronauts, can be caused by REP events. Ueno et al. (2019) quantitatively evaluated the exposure dose rate of REP events during extravehicular activity at the ISS, although the total effects are not severe even for the largest REP events.

At least two major types of plasma waves have been known and well-studied as the main causes of REP events: electromagnetic ion-cyclotron (EMIC) waves and whistler-mode chorus waves.

EMIC waves can resonate with MeV electrons as well as protons of tens of keV (e.g., Kubota et al. 2015; Miyoshi et al. 2008), scattering of the latter being able to produce so-called isolated proton aurora (IPA) (Sakaguchi et al. 2016). The expected emission altitude of the IPA ranges from 115 to 135 km (e.g., Liang et al. 2022; Shumko et al. 2022; Nakamura et al. 2022).

Chorus waves can also cause REP events. Chorus waves propagating toward the Earth along magnetic field lines can cause broadband electron precipitation ranging from a few keV to MeV leading to both diffuse aurora and REP events (Miyoshi et al. 2020). The chorus model has been confirmed by several conjunction observations between optical images from the ground and satellite/sounding rockets (Miyoshi et al. 2015; Kurita et al. 2015; Kawamura et al. 2021; Shumko et al. 2021; Namekawa et al. 2023). Although these observations were not directly related to the REP events, the emission altitude of such a diffuse aurora is well below 100 km (Brown et al. 1976; Hosokawa et al. 2024; Partamies et al. 2017).

Kataoka et al. (2016) first utilized the CALorimetric Electron Telescope (CALET) aboard the ISS to perform observations of the REP events. Since then, this instrument has been used to characterize the temporal and spatial properties of REP events (e.g., Kataoka et al. 2020; Bruno et al. 2022; Blum et al. 2024; Freund et al. 2024, 2025; Vidal-Luengo et al. 2024a, 2024b). Further, Zhang et al. (2025) conducted a statistical classification of the REP events driven by different causes such as chorus, EMIC, and field-line curvature scattering (FLCS).

While EMIC-driven REP events have been associated with proton aurora with emission altitudes exceeding 100 km, as expected from IPA (Liang et al. 2022), the emission altitude of aurora directly linked to chorus-driven REP events has not yet been quantitatively determined. In the case of broadband electron precipitation spanning energies from tens-of-keV to MeV driven by propagating chorus waves, the lower-energy electrons are expected to cause optical emission at below 100 km.

The purpose of this paper is to investigate auroral emissions observed near the ISS magnetic footprint during a REP event and to estimate their emission altitude using ground-based stereoscopic observations. By doing so, we also verify the effectiveness of altitude estimation as a diagnostic tool for better understanding the mechanisms of REP events and demonstrate that ground-based stereoscopic imaging can complement point-like satellite observations by providing additional spatial and temporal context. Section 2 describes the instrumentation and data used in this study. Section 3 explains the methods for identifying events in which auroral emissions were observed near the ISS magnetic footprint during REP events and estimating auroral emission altitudes. Section 4 presents the results of these analyses. Section 5 discusses the implications of the findings in the context of REP drivers and wave–particle interactions. Section 6 summarizes the conclusions of this study.

## Instrumentation and data

In this study, we investigated the characteristics and altitudes of auroral emissions observed near the ISS magnetic footprint during REP events using coordinated space- and ground-based observations using space-based measurements from the ISS and ground-based data from all-sky imagers installed in Athabasca, Canada. The ISS carries several scientific instruments capable of detecting energetic particles. Among them, CALET and Monitor of All-sky X-ray Image (MAXI) are suitable for identifying REP events (Kataoka et al. 2016).

### Instruments on the International Space Station (ISS)

CALET has been operating onboard the ISS since 2015 to measure high-energy cosmic-ray electrons and nuclei (Asaoka et al. 2018; Torii 2017). The instrument includes the Charge Detector (CHD), composed of segmented plastic scintillators arranged in two layers (CHD-X and CHD-Y) to measure the electric charge. The trigger counter signals from these layers are accumulated every 1 s. Although CHD was originally designed for cosmic-ray observations, its 1-s count rates can also be used to monitor MeV electron precipitation events in the radiation environment (Kataoka et al. 2016). In this study, we utilize the count rates of CHD-X and CHD-Y, which are sensitive to electrons with energies above 1.5 MeV and 3.4 MeV, respectively.

The Radiation Belt Monitor (RBM) of the MAXI instrument (Matsuoka et al. 2009) detects electrons and protons above 0.3 and 3 MeV, respectively, with a 1 s time resolution. Two identical sensors are installed: RBM-H, directed horizontally (limbward), and RBM-Z, directed vertically (zenith). RBM data therefore complement CALET by providing directional information and coverage of lower-energy precipitation (0.3–1.5 MeV).

Following the method of Bruno et al. (2022), REP events were identified based on the ratio of count rates in the two CHD layers: R_xy_ = N_x_ / N_y_ > 1 + 3σ_Rxy_ where N_X_ and N_Y_ denote 1-s count rates from CHD-X and CHD-Y, respectively, and σ_Rxy_ is the standard deviation of R_xy_ computed over 10-min time window. In addition, to confirm that the observed CHD ratio enhancements were caused by actual REP rather than orbital effects (e.g., variations associated with the ISS trajectory through the radiation belts), we imposed further criterion on the RBM data. The criterion is that the RBM-Z counts, sensitive to energetic electron precipitation, exhibit a burst-like increase relative to the RBM-H counts, as identified by visual inspection. Since RBM-Z is oriented perpendicular to the Earth's surface, an enhancement in RBM-Z relative to RBM-H indicates precipitation directed earthward, thus supporting the identification of REP.

### Ground-based all-sky camera observations in Athabasca

To investigate visible auroral emissions associated with REP events, we installed two identical all-sky cameras, ZWO ASI 183 mm Pro equipped with Fujinon fish-eye lens (FE185C086HA-1) at observation sites ~ 24.5 km apart, Athabasca University Geophysical Observatory (AUGO; 54.71°N, 113.31°W) and Athabasca University GeoSpace Observatory (AUGSO; 54.60°N, 113.64°W) in Athabasca, Canada. To control the camera, we use a single-board computer (Raspberry Pi 4). We set a 427.8-nm optical filter between the lens and sensor. The effective bandwidth (full width at half maximum; FWHM) of the filter was determined to be approximately 10 nm based on the angular dependence of the interference filter. The selected wavelength corresponds to N₂⁺ first negative band, because auroral emissions at this wavelength are mainly produced by precipitating electrons with energies of several tens of keV. We set the exposure time to be 18 s, with images taken every 20 s. The image acquisition timing of the two cameras was synchronized via NTP using the Raspberry Pi clocks. The timing accuracy is better than approximately 1 s, which is sufficient for this study.

This stereoscopic configuration, combined with long-exposure imaging and high-sensitivity optics, allows us to analyze faint diffuse auroral emissions at low brightness levels. The instrumental noise level was estimated from dark-frame measurements. The spatial standard deviation of the pixel counts in the central region was 357 counts for an exposure time of 18 s, corresponding to approximately 1.6 Rayleigh using a calibration factor of 0.0046 Rayleigh per count. The camera response was confirmed to be linear over the relevant radiance range in a laboratory calibration experiment. Here, this value represents the 1σ instrumental noise level under dark conditions. In actual observations, effective detectability depends on the sky background, which is non-zero even under clear and moonless conditions. The camera was configured with a 4 × 4 on-chip binning mode and an effective sensor ROI of 3648 × 3648 pixels, producing 912 × 912 pixel 16-bit images, although the original sensor resolution of the ASI183MM Pro is 5496 × 3672 pixels.

## Methods

### Selection and classification of REP events

From the available dataset, we selected conjugate REP events that met magnetic conjugacy with Athabasca observation sites by tracing the ISS orbit using the International Geomagnetic Reference Field (IGRF)-13 geomagnetic field model. We required that the magnetic footprints be observed with elevation angles greater than 30° from both all-sky imagers, and that the events also satisfied a temporal coincidence with auroral emissions observed by the cameras. We analyzed the REP events observed during the period from September 2024, when our observations started, to September 2025. We identified a total of 23 conjugate REP events, among which 10 occurred under clear-sky conditions. The observation of the remaining 13 REP events was affected by clouds, snow, or strong moonlight, preventing reliable auroral imaging. Among the selected 10 events, two REP events showed auroral emissions near the ISS footprint. Another five REP events exhibited auroral activity in regions spatially displaced from the footprint, while the remaining three REP events showed emission levels below 1.6 Rayleigh, due to low intensity or background noise.

### Estimation of auroral emission altitude

For analysis of stereoscopic observations from two all-sky cameras (AUGO and AUGSO) in Athabasca with overlapping all-sky fields of view, we use two different altitude estimation methods, which complement each other as explained below.

The first method is based on finding the geographical transform which gives the maximum correlation (Kataoka et al. 2013). We projected the all-sky images from both sites onto a horizontal plane at a series of assumed emission altitudes in geographic coordinates. We assumed emission altitudes in the range of 60–140 km with a step size of 5 km. For each assumed altitude, the same geographic region was projected onto each image using a linear scaling with altitude, ensuring that the corresponding area was compared between the two cameras. We divided the projected images into regions, and we calculated the cross-correlation coefficient between the image pairs from the two sites for each region.

**Fig. 1:**
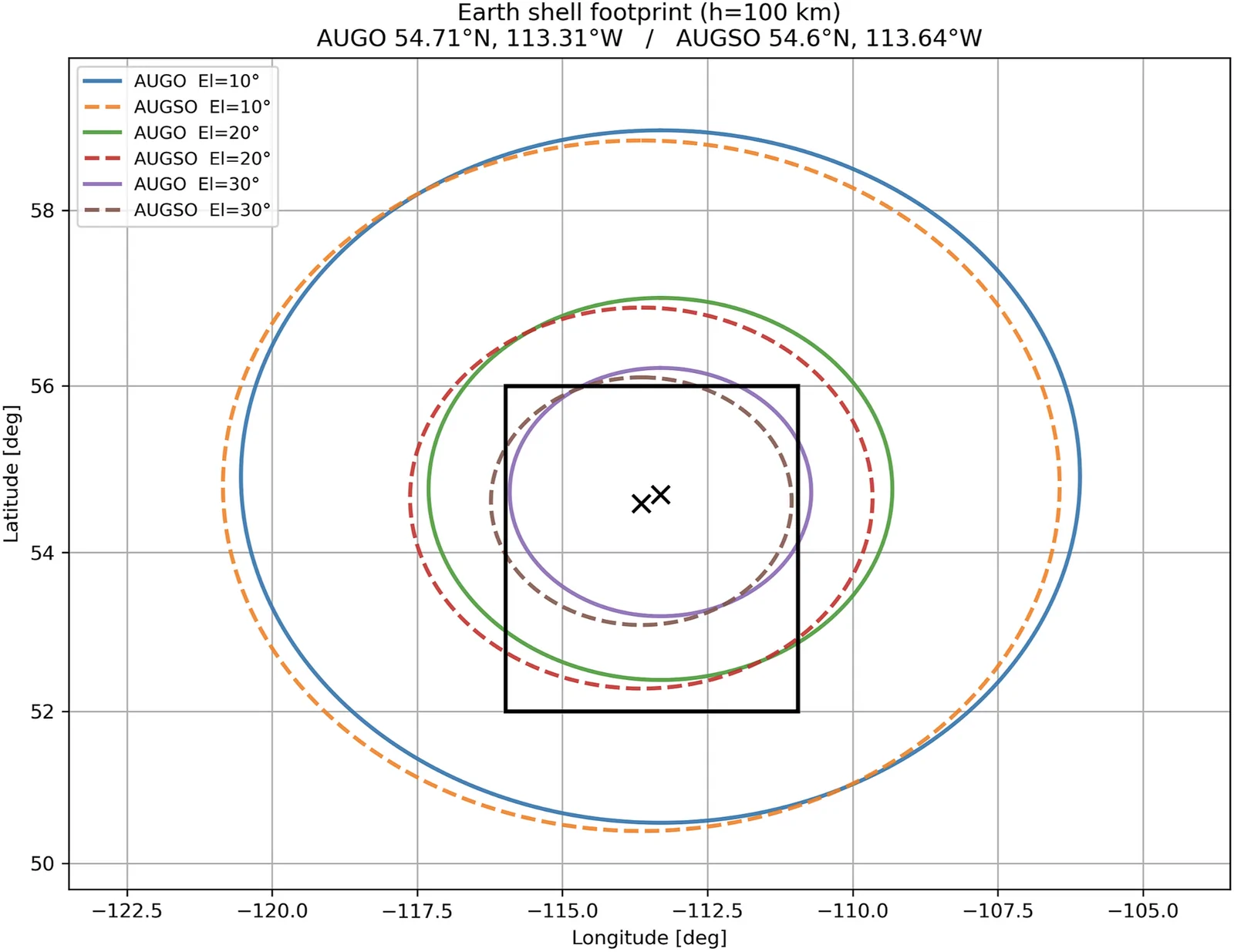
Geographic footprints of the fields of view of the two all-sky cameras (AUGO and AUGSO) projected onto a horizontal plane at an assumed emission altitude of 100 km. Solid and dashed curves represent AUGO and AUGSO, respectively, for different elevation angles (10°, 20°, and 30°). The black rectangle indicates the analysis region (52°–56° N, 111°–116° W), which was selected based on the overlap of the camera fields of view and the expected location of the ISS magnetic footprint. The southern boundary was extended to include possible auroral emissions near the ISS magnetic footprint

The correlation coefficient *r* was calculated as the Pearson correlation coefficient:

$$r\, = \,\sum {\left( {I_{1} \, - \,\overline{I}_{1} } \right)} \,\left( {I_{2} - \overline{I}_{2} } \right)\,/\,\left[ {\sigma \left( {I_{1} } \right)\sigma \left( {I_{2} } \right)} \right]$$

where *I*_1_ and *I*_2_ are the pixel intensities (in counts) of the two projected images within the overlapping region. The correlation was calculated on a pixel-by-pixel basis without spatial averaging. We interpreted the altitude that gave the maximum correlation as the auroral emission height. We applied this geographical transform method within a restricted region of 52°-56° N and 111°–116° W to minimize geographic distortion in the projection. This region was selected based on the overlap of the camera fields of view and the expected location of the ISS magnetic footprint (Fig. 1). While the northern boundary approximately corresponds to the region where both cameras observe above an elevation angle of 30°, the southern boundary was extended to lower latitudes to include possible auroral emissions near the ISS magnetic footprint. To ensure the reliability of the estimated emission altitude, we retained only the solutions that satisfied two quality criteria: (i) a peak correlation coefficient greater than 0.7, and (ii) a full width at 95% maximum of the correlation curve less than 50 km. These criteria allowed us to remove poorly constrained or multi-peaked solutions and ensured physically meaningful altitude estimates. We did not explicitly correct for atmospheric extinction and optical effects such as vignetting. The analysis is therefore restricted to a central region (elevation > 30°), where these effects are relatively small.

The second method is based on magnetic field-line tracing (Whiter et al. 2013): We traced geomagnetic field lines using the IGRF-13 model and overlaid them onto the all-sky images. For each field line, we identified the corresponding pixels and converted their values into auroral emission intensity in Rayleigh. The conversion was performed using a calibration factor derived from laboratory measurements. Before the conversion, we subtracted the mean dark count derived from a dark-frame image and applied a flat-field correction based on an integrating-sphere calibration image to account for spatial sensitivity variations across the field of view. We then calculated the emission intensity as I = α × C_{corr}, where C_{corr} is the dark-count- and flat-field-corrected pixel count, and α = 0.00446 Rayleigh per count. Then, assuming that the emission occurs along the field line, we constructed an altitude profile of aurora emission intensity by mapping the intensity as a function of altitude along each line. The emission altitude was calculated by averaging only the profiles that satisfied our selection criteria at the two observation sites. We imposed five selection criteria. These include: (1) agreement of peak altitudes at the two sites within ± 5 km; (2) exclusion of peaks occurring within 10 km of the prescribed altitude bounds to avoid edge effects, i.e., artificial truncation of the emission profile near the upper and lower altitude bounds; (3) consistency of emission centroids within 20 km; (4) a correlation coefficient greater than 0.8 between the two Rayleigh–altitude profiles; and (5) a full width at 90% maximum of the correlation curve less than 50 km to ensure a well-defined emission layer.

The geographical transform method is most effective for diffuse aurora near the magnetic zenith, where auroral structures are thin and horizontally extended. However, this technique is affected by geographic distortion and limited spatial resolution, making it unsuitable for tall discrete aurora especially near the edge of all-sky field-of-view. On the contrary, the field-line tracing method is more appropriate for discrete aurora with well-defined vertical structures, even for auroral forms at low elevation angles.　Nevertheless, this method becomes unreliable for thin diffuse aurora near the magnetic zenith, as such emissions do not show distinct ray structure associated with magnetic field lines. In both methods, the altitude step size is 5 km, which gives a discretization uncertainty of ± 2.5 km in the peak altitude. The auroral emission extends over a finite vertical range. We interpret the peak altitude as a representative value of the emission layer rather than a single well-defined altitude. This interpretation reflects the width of the correlation peak and the spread of the Rayleigh–altitude profiles, which limit the identification of a unique emission height. By combining the two approaches, we can obtain a more reliable altitude estimate across the entire all-sky field-of-view, for both types of auroral morphology.

## Results

**Fig. 2:**
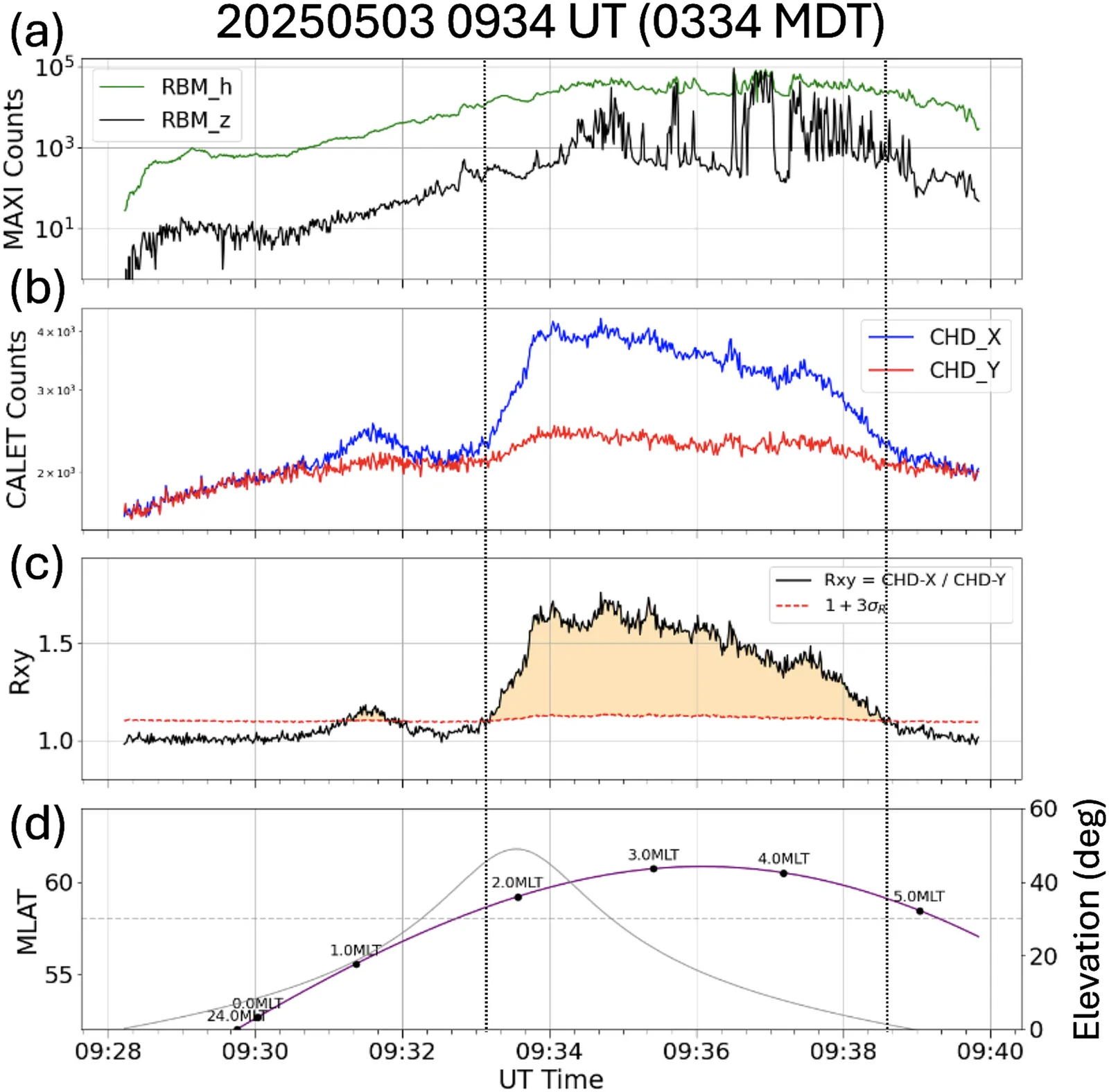
Time series of the REP event observed on 3 May 2025 from 09:28 to 09:40 UT: **a** RBM-Z and RBM-H count rates from MAXI, **b** CHD-X and CHD-Y count rates from CALET, **c** the ratio of count rates in the two CHD layers R_xy_, and **d** the ISS sub-satellite magnetic latitude (MLAT) and magnetic local time (MLT), together with the elevation angle of the ISS as viewed from AUGSO (right axis), calculated from the ISS position data

An overview of the selected REP event is summarized in Fig. 2. In the top panel, the REP event appeared on 3 May 2025 between 09:33 and 09:39 UT, when the ISS footprint moved from MLAT 48° to 60° across the dawn side sector (01–04 MLT) as shown in the bottom panel. The CHD-X counts began to increase at 09:33:00 UT, reached a maximum of ~ 4.0 × 10^3^ counts s⁻^1^ at 09:34:00 UT, and gradually decreased until 09:37 UT before dropping sharply to the CHD-Y level (~ 2.0 × 10^3^ counts s⁻^1^) by 09:39 UT. The R_xy_ (= CHD-X counts / CHD-Y counts) reached ≈ 2.0 at 09:34:30 UT, and the CHD-Y counts remained nearly constant around ~ 2.0 × 10^3^ counts s⁻^1^ throughout the interval.

The RBM-Z channel began to increase at 09:34:00 UT, about 30 s after the CHD-X increase, and showed multiple burst-like spikes between 09:34 and 09:37 UT. The count rate increased from ~ 10^2^ to 10^4^ s⁻^1^. The amplitude of the short spikes, each lasting a few seconds, became largest at 09:36 UT, when the RBM-Z/RBM-H count ratio ≈ 1.0. The RBM-H channel maintained a high baseline (10^4^–10^5^ counts s⁻^1^) and gradually increased over the same time interval.

**Fig. 3:**
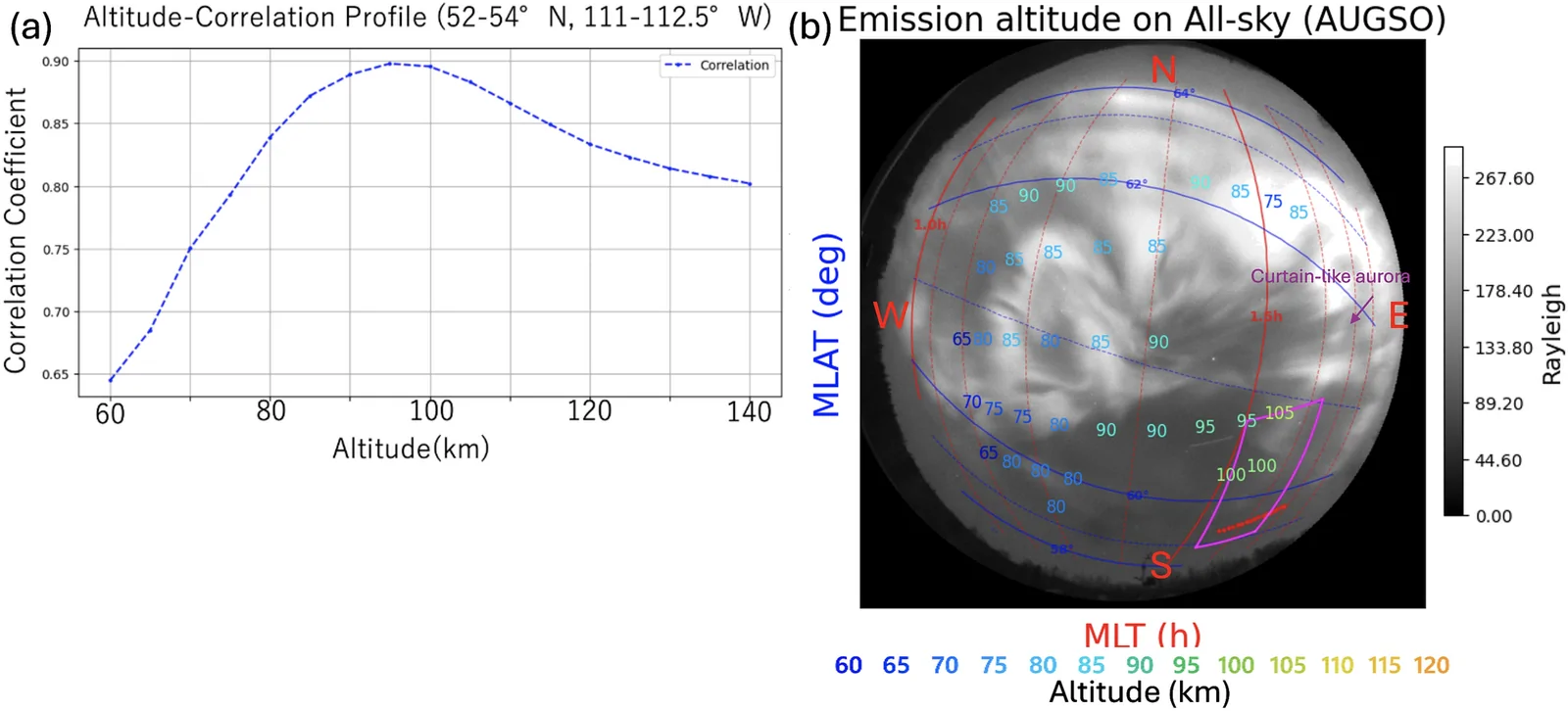
Geographical transform analysis of the 427.8 nm auroral emission observed at 09:33:40 UT on 3 May 2025, showing **a** the image-correlation coefficients between AUGO and AUGSO as a function of assumed emission altitude (60–140 km) of aurora in the magenta-boxed region of (b) (52°–54° N, 111°–112.5° W), and **b** an AUGSO all-sky image mapped emission altitude obtained by the geographical transform method. The projected all-sky images were divided into 1° × 1° regions, sampled every 0.5° in both latitude and longitude, and the correlation coefficient was calculated for each region. Solid red (dashed) curves denote major (minor) MLT isolines, and solid (dashed) blue curves denote major (minor) MLAT isolines. The red dashed line shows the ISS magnetic footprint, and the magenta frame the analysis region used in panel (**a**). The grayscale color bar represents the auroral intensity in Rayleigh. The arrow indicates the curtain-like auroral structure discussed in the text. Longitudes are given in degrees west, and the map is oriented with north upward and east to the right

Count rates in CHD-X and RBM-Z exhibited a rapid increase within approximately one minute around 09:33 UT and returned to their background levels by 09:39 UT, when the ISS footprint was located near MLAT ≈ 58°. Figure 3a shows the altitude–correlation profile derived from the geographical transform method applied to the auroral images at 09:33:40 UT. We computed the correlation in the region indicated by the magenta frame in Fig. 3b (52-54N, 111–112.5W, defined assuming an emission altitude of 100 km). The profile exhibits a clear peak around 95 km, and we interpret this altitude as the most probable emission height of the diffuse aurora inside the analysis region.

Figure 3b summarizes the spatial distribution of the inferred emission altitude. The overplotted MLT and MLAT isolines (red and blue) help to identify the geomagnetic context of the emission. The analyzed region lies near ~ 1.2–1.6 MLT and MLAT 59° to 63° at this event. The red dashed line marks the ISS magnetic footprint, which passed near 52.5N, 112W ~ 113W (~ 1.6 MLT and MLAT ~ 59°) during these 20 s (09:33:40 ~ 09:34:00 UT). The ISS magnetic footprint (red dotted line) passed approximately 50–60 km from the equatorward edge of the diffuse aurora, assuming an emission altitude of 100 km from the angular separation in latitude. The auroral emission was spatially extended and modulated. This image shows the best condition in which the ISS passed over the structured aurora at a relatively high elevation angle from the ground-based observation sites. We do not observe a latitudinal trend within the analysis region. However, the emission altitude increased slightly from west to east (from ~ 70–90 km to ~ 90–100 km). While the western part of the analysis region is dominated by patchy diffuse structures, the eastern part includes more pronounced curtain-like aurora (indicated by arrows in Fig. 3b), which likely contributes to the slightly higher inferred altitudes on the east side.

**Fig. 4:**
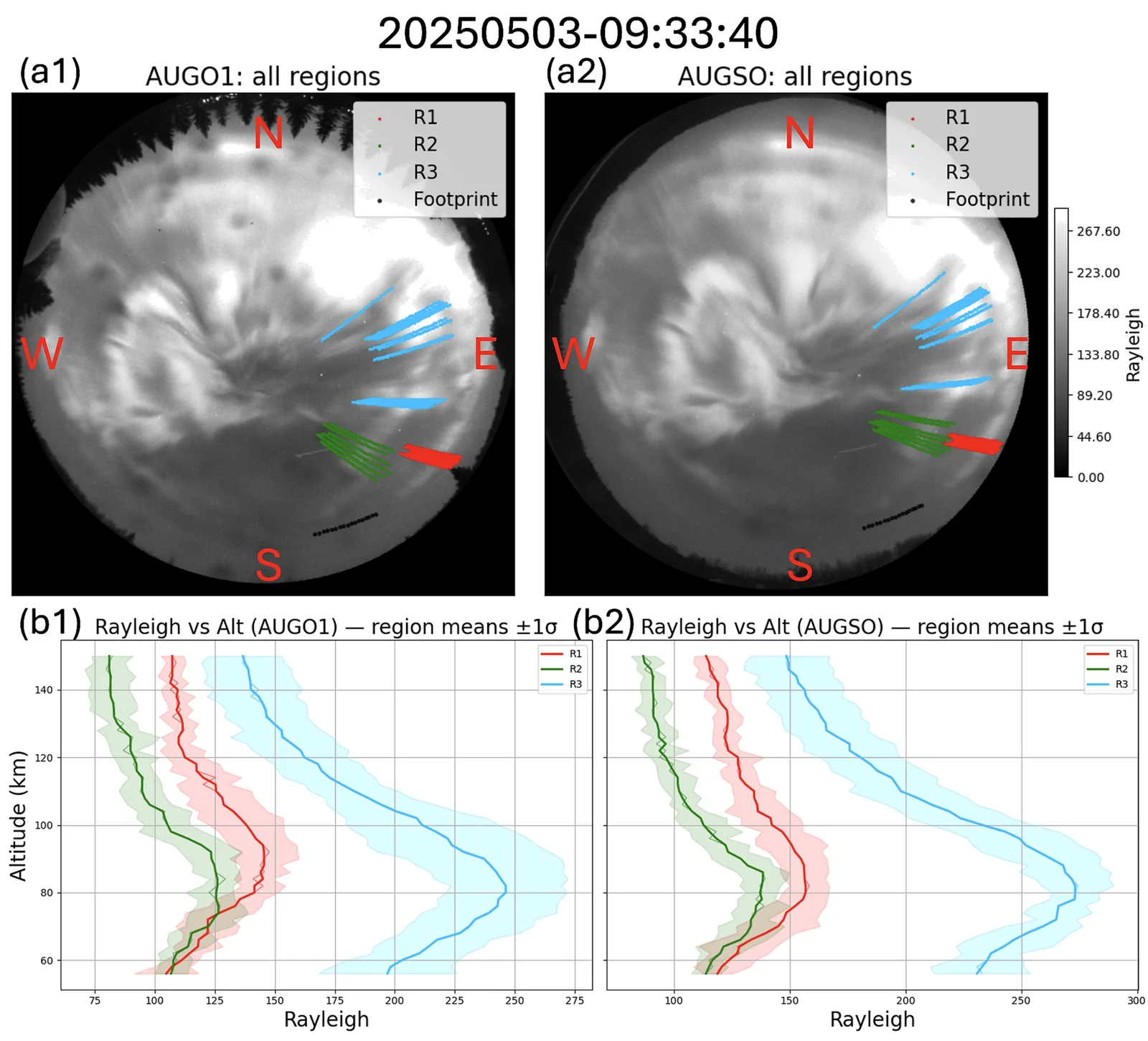
Field-line tracing analysis of the 427.8-nm auroral emission observed at 09:33:40 UT on 3 May 2025. **a** All-sky images mapped along magnetic field lines that satisfied all selection criteria, grouped into three analysis regions: R1 (red; 52°–53° N, 109°-111° W), R2 (green; 53°–54° N, 111°-113° W), and R3 (blue; 54–55° N, 111°-113° W). The black dashed line shows the ISS magnetic footprint. **b** The mean Rayleigh–altitude profiles (± 1σ) derived from the accepted field lines in each region

Figure 4 shows the results from the magnetic field-line tracing method. In Fig. 4a, only the field lines that satisfied all criteria in the profiles obtained from AUGSO and AUGO are depicted. Different colors denote the eastern-side field lines in different regions R1–R3. We analyzed magnetic field lines traced over the southeastern part of the image (52°–55° N, 109°–112.5° W). On the western side of the aurora, we do not identify any field lines that meet our criteria, possibly because patchy and horizontally extended structures dominate the morphology and fail to produce well-defined peaks in the Rayleigh–altitude profiles. Figure 4b shows the averaged emission altitude profile of the eastern-side field lines in regions R1–R3 shown in Fig. 4a. The obtained peak emission altitudes are ~ 95 km in R1 (52°-53°N, 109°-111°W), ~ 85 km in R2 (53°-54°N, 111°-112.5°W), and ~ 80 km in R3 (54°-55°N, 111°-112.5°W). The emission layer extends over ~ 15 km in altitude below 100 km, consistent with the optical emissions produced by tens-of-keV electron precipitation. From these two different methods, we conclude that the emission altitude is a representative value of 80–95 km during this particular REP event.

**Fig. 5:**
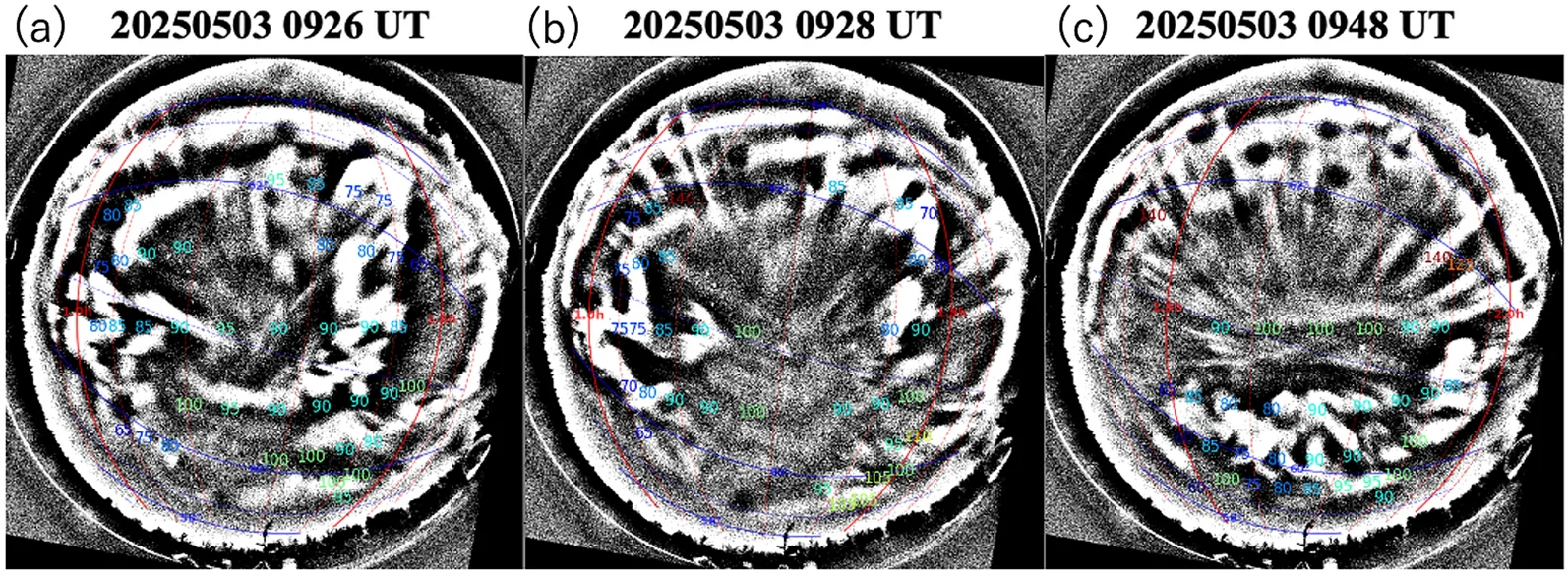
Snapshots of auroral emission altitude maps derived using the geographical transform method at three selected times on 3 May 2025: **a** 09:26 UT, **b** 09:28 UT, and **c** 09:48 UT. The projection is the same as in Fig. 3b. Solid red (dashed) curves indicate major (minor) magnetic local time (MLT) isolines, and solid (dashed) blue curves indicate major (minor) magnetic latitude (MLAT) isolines

Figure 5 shows the auroral emission altitude maps from the geographical transform method at three different times on 3 May 2025: 09:26 UT, 09:28 UT, and 09:48 UT. These maps show both temporal and spatial variations in the estimated emission altitudes. Notably, regions exhibiting patchy aurora—compact and well-defined structures—tend to show lower emission altitudes, typically in the range of 70–85 km. In contrast, areas appearing as diffuse aurora are associated with higher altitudes, generally around 90–100 km. This trend suggests that auroral morphology has a correlation with the estimated altitude. At 09:26 UT, patchy aurora dominated both the western and eastern regions, whereas the southern and near-zenith areas displayed less structured auroral forms, corresponding to higher estimated altitudes. A similar pattern was observed at 09:28 UT, where patchy aurora in the western and northeastern sectors were associated with lower altitudes, while more diffuse aurora in the southeastern region exhibited higher emission altitudes. At 09:48 UT, the region near the zenith became dominated by diffuse aurora, leading to higher altitude estimates compared to the more structured patchy aurora in the southwestern to southern areas. Different auroral morphologies appear in regions with different ranges of estimated emission altitudes. Values of ~ 65–70 km and ~120–140 km are also present, mainly near the edge of the field of view. Because these extreme values occur near the field-of-view edge, where the altitude estimation is less reliable, they should not be interpreted as precise emission heights. Instead, they indicate the possible lower and upper extent of the inferred emission layer in this event.

## Discussion

In this study, we installed a new stereoscopic all-sky imager system for auroral emissions observed near the ISS magnetic footprint during REP events as observed by CALET onboard ISS. The results shown above can be summarized as follows: we identified a REP event on 3 May 2025, which showed electrons in the 0.3–3.4 MeV energy range precipitating along magnetic field lines crossed by the ISS orbit between 09:33 and 09:39 UT (Fig. 2). Using ground-based optical observations at Athabasca, the ISS magnetic footprint passed approximately 50–60 km from the diffuse auroral region (Fig. 3b), assuming an emission altitude of 100 km. Using the geographic transform method, we estimated the emission altitude of the diffuse aurora to be ~ 95 km (Fig. 3a). Using the magnetic field-line tracing method, we obtained consistent results (Fig. 4), typically associated with tens-of-keV electron precipitation (Rees et al. 1963). In addition, the auroral emission altitude did not show a clear latitudinal dependence within the analysis region (Figs. 3b and 4b), while a slight longitudinal variation was observed within the analysis region (Fig. 3b), with lower altitudes in the west and higher altitudes in the east. Figures 3 and 5 show that different auroral morphologies appear in regions with different ranges of estimated emission altitudes.

Note first that identifying a chorus-related auroral emission itself is not a new finding. There are many examples of pulsating auroras, which are associated with chorus waves. Kurita et al. (2015) identified REP event-related pulsating auroras and demonstrated the link between chorus-driven precipitation and auroral modulation. As also summarized in the Introduction, the emission altitude of auroral emissions observed near the ISS magnetic footprint provides a diagnostic for the wave–particle interaction type: chorus-driven REP events tend to produce auroral emissions below 100 km, whereas EMIC-driven REP is typically linked to aurora emissions above 100 km. The results obtained in this study are consistent with the former case (Kurita et al. 2015), suggesting that the analyzed event was chorus-driven REP events. The key advance of this study is that we determined the emission altitude of the aurora observed within approximately 50–60 km of the ISS magnetic footprint, using two independent methods.

We confirmed the corresponding REP events are in the MeV and sub-MeV range, and we identified that the auroral emission observed within approximately 50–60 km of the ISS magnetic footprint is consistent with emission from tens-of-keV electrons. The broadband electron precipitation, spanning tens of keV to a few MeV, can be a consequence of electron resonance with duct-propagating chorus waves (Miyoshi et al. 2020).

The altitude estimates in this study have several limitations that affect their interpretation. In the geographical transform method, the accuracy decreases near the edge of the field of view, where the effective parallax between the two cameras becomes smaller and geometric errors become larger. In the field-line tracing method, the auroral emission extends over a broad region, and multiple structures can contribute to the signal along the same line of sight, particularly near the edge of the field of view. In such a geometry, emission from higher altitudes can be projected onto lower apparent altitudes, which can bias the estimated peak altitude toward lower values. These effects limit the ability to interpret small differences in the estimated altitude. The estimated altitudes should be regarded as approximate indicators of the emission layer rather than precise altitude determinations.

We found no systematic latitudinal variation in the auroral emission altitude. FLCS is expected to produce energy-dispersed precipitation, in which higher-energy electrons precipitate at lower magnetic latitudes and therefore generate lower emission altitudes toward the equator (Sivadas et al. 2019; Murase et al. 2022). Such a signature was not observed in our data. Instead, the altitude remained nearly uniform at ~ 90 km over a horizontal scale of approximately 400 km in latitude (52–56°N), indicating that tens-of-keV electrons precipitated. The observed altitude distribution does not show the latitudinal trend expected from FLCS-driven precipitation and is more consistent with chorus-driven broadband precipitation.

In this event, patchy aurora, characterized by compact and well-defined structures, tend to occur at lower altitudes (70–85 km), while diffuse aurora—appearing more blurred or faint—are associated with higher emission altitudes (90–100 km). However, the present analysis does not establish a systematic relationship between auroral morphology and emission altitude. Small differences in the estimated altitude cannot be clearly distinguished within the present analysis, and the altitude estimation becomes less reliable when the auroral morphology is ambiguous or when the emission is faint. The apparent relationship between auroral morphology and estimated emission altitude is partially affected by the spectral sensitivity of the imaging system. The 427.8 nm wavelength is primarily sensitive to high-energy electron precipitation (tens of keV), and emissions driven by lower-energy electrons (a few keV), which typically occur at higher altitudes, are less efficiently detected at this wavelength. As a result, such aurora appear more diffuse simply due to reduced optical response—introducing an apparent “morphology–altitude” effect shaped by observational bias. Therefore, the differences between diffuse and patchy aurora observed in this event may partially reflect observational bias associated with the single-wavelength imaging system. Future studies will pursue coordinated multi-wavelength observations and analyses of multiple events to improve the accuracy of morphological classification and altitude estimation, and to better resolve the underlying energy characteristics of precipitating particles.

We also demonstrate that auroral emissions observed near the ISS magnetic footprint during REP events are not frequent, based on a systematic survey of conjugate REP events. In our year-long observation period, we found that only two out of ten conjugate REP events exhibited visible aurora within approximately 50–60 km of the magnetic footprint.

The use of a 427.8-nm optical filter is one possible reason to understand the infrequent occurrence of auroral emissions observed near the ISS magnetic footprint. Note that the 557.7-nm oxygen line basically provides the strongest optical signature of EMIC-related proton precipitation (Sakaguchi et al. 2007; Liang et al. 2022), whereas the 427.8-nm emission used in this study has a weaker response to the proton aurora. Given that our imager system is therefore less sensitive to the EMIC-related proton aurora, our findings that only two out of 10 REP events showed auroral emissions near the ISS magnetic footprint, and both of those events are likely chorus related, is not inconsistent with the recent statistical result of Zhang et al. (2025), where they noted that EMIC-related REP events are comparable or more frequent than chorus-driven REP events.

We modernized the ground-based experimental setup by utilizing a low-cost single-board computer for controlling the all-sky camera, also setting an optical filter between the lens and sensor to make the imager smaller. We demonstrated that the compact and twin imager systems can effectively complement satellite measurements by providing quantitative information on auroral emission altitudes as well as the 2D spatial context which is complementary to in situ satellite observations. For future work, we aim to further reduce the cost without losing the sensitivity, to expand the all-sky observation network, hopefully via the spreading help of citizen-science participation (Kataoka et al. 2024; 2025).

## Conclusions

We analyzed the REP event on 3 May 2025 and identified MeV and sub-MeV electron precipitation with diffuse aurora observed near the ISS magnetic footprint. We modernized the stereoscopic all-sky camera observations, then we estimated the auroral emission altitude to be ~ 90 km, which is typically due to tens-of-keV electrons. Such broad-energy observations from tens-of-keV to MeV are consistent with chorus-driven REP events, and the observed altitude distribution does not show the latitudinal trend expected from FLCS-driven precipitation. Our results demonstrate that compact and low-cost all-sky imagers can effectively complement the in situ satellite observations by providing auroral altitude and spatial context.

## Data Availability

The datasets analyzed in this study are publicly available through the Data Archives and Transmission System (DARTS) operated by the Institute of Space and Astronautical Science (ISAS), Japan Aerospace Exploration Agency (JAXA). The CALET Charge Detector (CHD) Level 1.1 data are available at: https://darts.isas.jaxa.jp/pub/calet/cal-v1.1/CHD/level1.1/obs/2025/. The MAXI Radiation Belt Monitor (RBM) data are available at: https://darts.isas.jaxa.jp/pub/maxi/rbm/2025/**.** Ground-based auroral images obtained at Athabasca are not publicly archived due to limited storage and data privacy policies, but they are available from the corresponding author upon reasonable request. The raw 16-bit all-sky image data used in this study, including the images corresponding to Figs. 3, 4, 5, are publicly available at Zenodo: https://doi.org/10.5281/zenodo: https://doi.org/10.5281/zenodo.20110634.

## Abbreviations

REP: Relativistic electron precipitation
EMIC: Electromagnetic ion-cyclotron
IPA: Isolated proton aurora
CALET: CALorimetric Electron Telescope
CHD: CHarge Detector
MAXI: Monitor of All-sky X-ray Image
RBM: Radiation Belt Monitor
ISS: International Space Station
LEO: Low-Earth orbit
FLCS: Field-line curvature scattering
IGRF: International Geomagnetic Reference Field
